# Electrothermally Driven Reconfiguration of Microrobotic Beam Structures for the ChipSail System

**DOI:** 10.3390/mi14040831

**Published:** 2023-04-09

**Authors:** Kecai Xie, Chengyang Li, Shouyu Sun, Chang-Yong Nam, Yong Shi, Haipeng Wang, Wu Duan, Zhongjing Ren, Peng Yan

**Affiliations:** 1 Key Laboratory of High-Efficiency and Clean Mechanical Manufacture, Shandong University, Jinan 250061, China; 2 Center for Functional Nanomaterials, Brookhaven National Laboratory, Upton, NY 11973, USA; 3 Department of Mechanical Engineering, Stevens Institute of Technology, Hoboken, NJ 07030, USA; 4 Department of Endocrinology, Qilu Hospital of Shandong University, Jinan 250012, China

**Keywords:** solar sail spacecraft, chip-scale satellite, bilayer beam, electrothermal reconfiguration, ChipSails

## Abstract

Solar sailing enables efficient propellant-free attitude adjustment and orbital maneuvers of solar sail spacecraft with high area-to-mass ratios. However, the heavy supporting mass for large solar sails inevitably leads to low area-to-mass ratios. Inspired by chip-scale satellites, a chip-scale solar sail system named ChipSail, consisting of microrobotic solar sails and a chip-scale satellite, was proposed in this work. The structural design and reconfigurable mechanisms of an electrothermally driven microrobotic solar sail made of Al\Ni_50_Ti_50_ bilayer beams were introduced, and the theoretical model of its electro-thermo-mechanical behaviors was established. The analytical solutions to the out-of-plane deformation of the solar sail structure appeared to be in good agreement with the finite element analysis (FEA) results. A representative prototype of such solar sail structures was fabricated on silicon wafers using surface and bulk microfabrication, followed by an in-situ experiment of its reconfigurable property under controlled electrothermal actuation. The experimental results demonstrated significant electro-thermo-mechanical deformation of such microrobotic bilayer solar sails, showing great potential in the development of the ChipSail system. Analytical solutions to the electro-thermo-mechanical model, as well as the fabrication process and characterization techniques, provided a rapid performance evaluation and optimization of such microrobotic bilayer solar sails for the ChipSail.

## 1. Introduction

Diverse microstructures with high area-to-mass ratios (or low areal densities) have been developed and applied in a wide range of applications, including robotics [1,2,3], soft electronics [4,5], medical devices [6,7], and solar sailing in space [7,8,9,10,11]. Spacecraft equipped with proper solar sails can make good use of sunlight for photon propulsion once being released and deployed into space without consuming any fuel. The most recent and rapid development of robotic systems for active control of such solar sail spacecraft makes photon propulsion in space more appealing to researchers, and a series of engineering and space experiments of spacecraft with large solar sails for propellant-free solar sailing was completed. Some typical solar sail spacecraft, such as Cosmos-1 [12], IKAROS [9], NanoSail-D [8], and LightSails [13], demonstrated the feasibility of the approach toward building large solar sails via coating ultrathin aluminum membranes (20~70 nm) onto polymer films. Unfortunately, due to the heavy satellite body and additional supporting frames for the solar sails, the maximum acceleration that such a sail spacecraft can acquire after their sails’ deployment in low earth orbit is about 10^−5^ g [11], where g is the gravity at the orbital altitude of the spacecraft. While NASA and Planetary Society researchers have been working to minimize the thickness of the polymer sail and reduce the mass of the support arm or tip mass, the miniaturization of satellite bodies seems to be a more attractive solution to the development of the next generation of solar sail spacecraft with higher area-to-mass ratios and more efficient solar-sailing-based orbital maneuvering and attitude adjustment. Although the creation of chip-scale spacecraft with milligram-scale masses is available, the development and experimental validation of active and reconfigurable micro solar sails for a microrobotic solar sailing system is yet to be reported.

Atchison et al. investigated the influence of length scaling on the area-to-mass ratio of solar sail spacecraft [14], and a passive solar-pointing millimeter-scale solar sail with a thickness of 25 μm and a mass of 7.5 mg was developed through microfabrication technologies [15]. Boschetto et al. designed a new self-deploying solar sail system that was activated with a shape memory alloy wire with a diameter of 0.41 mm using an aluminum film with a thickness of 12 μm and an area of 100 cm^2^, where deployment tests were accomplished in an orbiting laboratory environment [16,17]. The aforementioned studies suggested that solar sails for chip-scale satellites could be built via microfabrication, and the expected area-to-mass ratios of such self-supported solar sail microstructures were up to 100 m^2^/kg [18], theoretically contributing to solar radiation accelerations up to 10^−3^ m/s^2^. Candidate driving methods for these micro solar sails include electric heating [19,20,21], electrostatic drives [22,23], electromagnetic actuation, and piezoelectric and shape memory effects [24,25,26]. Electrothermal actuation of bilayer metallic microstructures consisting of two metals with a larger difference in coefficients of thermal expansion was shown to be a feasible approach by our group [27,28,29,30,31]. We previously proposed a novel conceptual design of micro solar sails for a chip-scale spacecraft with a sub-gram mass referred to as ChipSail. The solar sail of the ChipSail could be developed based on an Al\Ni_50_Ti_50_ two-layer grid microstructures created via surface microfabrication. We theoretically established an electro-thermo-mechanical model of the ChipSail in low earth orbit, which allowed for the efficient prediction and evaluation of its reconstruction behaviors driven by Joule heating [28,29,30]. Furthermore, in this work, we report the progress of the chip-scale spacecraft design and experimental validation of the microrobotic solar sails of the ChipSail system for space exploration.

The rest of this article is organized as follows. First of all, the working mechanism of the electrothermally driven bilayer solar sails of the ChipSail system is described. Next, an electro-thermo-mechanical model of the reconfiguration of such solar sails in low Earth orbit is established for the displacement and bending angle of the solar sail under Joule heating. Then, the fabrication of proposed micro solar sails and experimental validation of their reconfiguration for microrobotic ChipSails is discussed. Finally, the results from theoretical models and experiments are discussed and conclusions are given.

## 2. Design of Microrobotic ChipSail System

**Figure 1:**
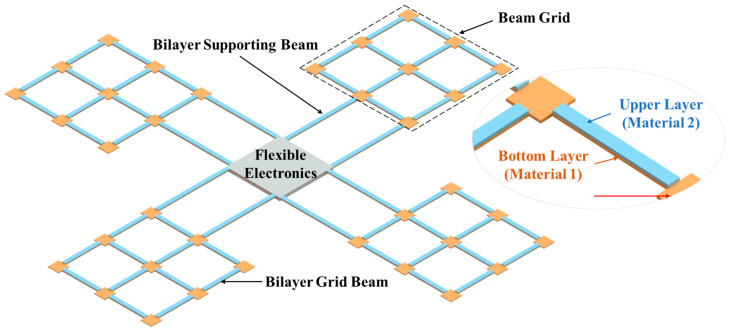
Conceptual design of the microrobotic ChipSail system for solar sailing (not to scale).

In this section, the structural design and working mechanism of the bilayer micro solar sail structure for the microrobotic ChipSail system are presented. A previous conceptual ChipSail design theoretically demonstrated the reconfigurability of the two-dimensional Al\Ni_50_Ti_50_ bilayer microstructure [18], which was attributed to the significant thermal mismatch between the Al and austenite Ni_50_Ti_50_. A new conceptual design of the ChipSail based on bilayer grid microstructures is further given in Figure 1. Compared with the previous one [18], this new concept shows great potential for the miniaturization of the satellite body (or flexible electronics) for the same-sized solar sails.

Generally, the complete ChipSail system herein is composed of a flexible electronics-based satellite body and four microrobotic solar sails capable of active reconfiguration. Reconfigurable behaviors of such microrobotic solar sails were mainly focused on in this work. As can be seen from Figure 1, each solar sail consisted of two layers with different materials. Al was selected as material 1, which was for the bottom layer, while Ni_50_Ti_50_ was chosen as material 2, which was for the upper layer. As such, there were two bilayer supporting beams and twelve bilayer grid beams in each bilayer solar sail, which could be electrothermally driven on the ground and in space. When there is a potential difference between the two supporting beams of a solar sail, a current will be generated across the conductive bilayer microstructure, contributing to distributed Joule heating of the bilayer solar sail, as well as significant out-of-plane thermo-mechanical deformation of the solar sail. Active reconfiguration of the solar sails enables adjustment of the solar pressure distribution across the reflective Al beam by tuning the incident angles of the sunlight on the reflective surface. It is worth pointing out that post-annealed Ni_50_Ti_50_ layers can have transformation temperatures that are much lower than ambient temperatures (20 °C), which implies that the post-annealed Ni_50_Ti_50_ layers are in a superplastic austenite phase. The coefficient of thermal expansion of the Ni_50_Ti_50_ layers was defined as 11 × 10^−6^ K^−1^, whereas that of the Al layers was set to 23.1 × 10^−6^ K^−1^ [32,33,34]. In addition, due to the good electrical conductivity of these two materials, the electro-thermal drive of thermo-mechanical deformation can be achieved via Joule heating, which was shown to be effective in previous studies [35,36,37].

Prior to the determination of the structural parameters of the microrobotic solar sail, it was necessary to identify the minimum requirements of the area-to-mass ratio of the microrobotic ChipSail. In this work, for simplification purposes, it was assumed that the mass of the flexible electronics-based satellite body was the same as the total mass of the four surrounding solar sails. According to previous studies, to realize efficient solar sailing using the microrobotic ChipSail system, its area-to-mass ratio should be above 100 m^2^/kg.

The area-to-mass ratio of the Al\Ni_50_Ti_50_ bilayer micro solar sail without a satellite body can be written as

(1) $$\frac{A}{m_{s}}=\frac{1}{\rho_{Al}t_{Al}+\rho_{NiTi}t_{NiTi}}$$

Given the hypothesis of $m_{s}=m_{c}$, the area-to-mass ratio of the whole ChipSail system with a mass of $m=m_{s}+m_{c}$ is modeled as

(2) $$\frac{A}{m}=\frac{1}{{2(\rho }_{Al}t_{Al}+\rho_{NiTi}t_{NiTi})}$$

It can be safely derived from Equation (2) that the area-to-mass ratio is mainly dominated by the thicknesses of the Al and Ni_50_Ti_50_ layers. Although the area-to-mass ratio can be increased by decreasing the corresponding thicknesses, excessive reduction in the thickness would inevitably lead to fatal failures. As such, the determination of these thicknesses is a compromise solution that takes both the strength and solar sailing requirements into consideration. Generally, the thickness of each layer in the grid microstructure is determined by two factors. On one hand, the bending stiffness of the sail should be high enough to alleviate the influence of the solar radiation pressure on the bending deflection of the sail. On the other hand, to ensure efficient photon propulsion, the area-to-mass ratio should be larger than 100 m^2^/kg [38], which means the thickness of each layer should be smaller than 1 μm. However, deposited layers with too small of a thickness would inevitably lead to structural faults, such as voids or vacancies. For example, Ni_50_Ti_50_ layers thinner than 0.2 μm can hardly function as shape memory alloys. Therefore, the thickness of 0.3 μm was used for the Ni_50_Ti_50_ layer. Given the bending stiffness match between the Al and Ni_50_Ti_50_, the thickness of Al was selected to be 0.3 μm. Hence, a typical set of parameters of the novel ChipSail concept is summarized in Table 1.

## 3. Electro-Thermo-Mechanical Model

### 3.1. Electro-Thermal Model

In this section, electro-thermo-mechanical behaviors of proposed micro solar sails in ambient temperature (20 °C) and high vacuum surroundings are analytically modeled, which can be divided into an electro-thermal model and a thermo-mechanical model. As is well known, convection, conduction, and radiation are the three modes of heat flow. Heat transfer due to convection and radiation was shown to be negligible in the condition considered in this work, while the heat transfer due to conduction between the solar sails and anchored substrates is the dominant factor [18]. Therefore, the thermal energy for actuation is provided only via Joule heating, while the energy outflow is determined by thermal conduction from the suspended bilayer solar sails to the substrate.

**Figure 2:**
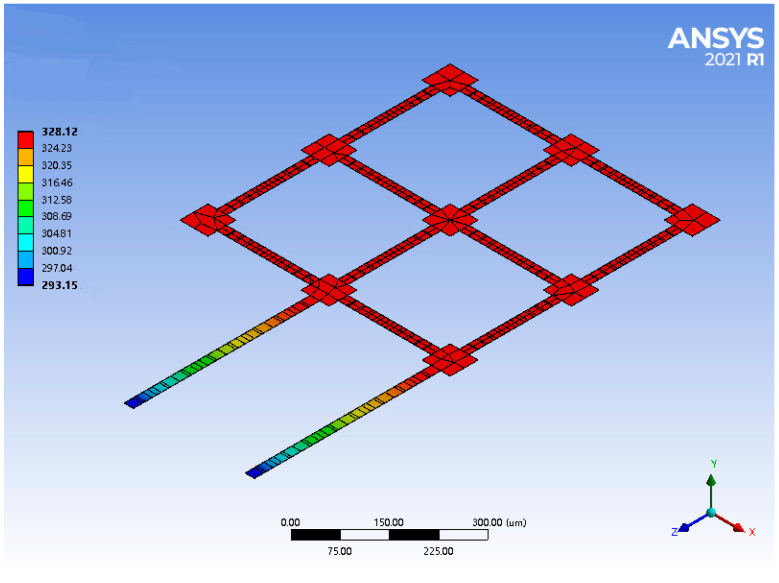
FEA results of temperature distribution across the bilayer solar sail by applying a voltage of 0.04 V.

Finite element methods can help to predict the behavior of products affected by Joule heating, and thus, the temperature distribution on the microstructure was performed by using Ansys 2021 (student version), which is professional FEA software. The mesh of the finite element model was acquired using the Sweep method with an element size of 3 μm. Given that the materials of Al and austenite Ni_50_Ti_50_ are elastic and isotropic, the contact type of the interface between the materials was set to be bonded in the FEA, which means that there was no relative sliding between the Al and austenite Ni_50_Ti_50_ layers. The geometrical data and mechanical properties of the bilayer micro solar sail, which were used for analytical modeling and finite element analysis, are listed in Table 1. As a result, the temperature distribution across the solar sail was acquired with a range of [293.15, 328.12] K by applying a constant voltage of 0.04 V between the supporting beams, as shown in Figure 2.

**Figure 3:**
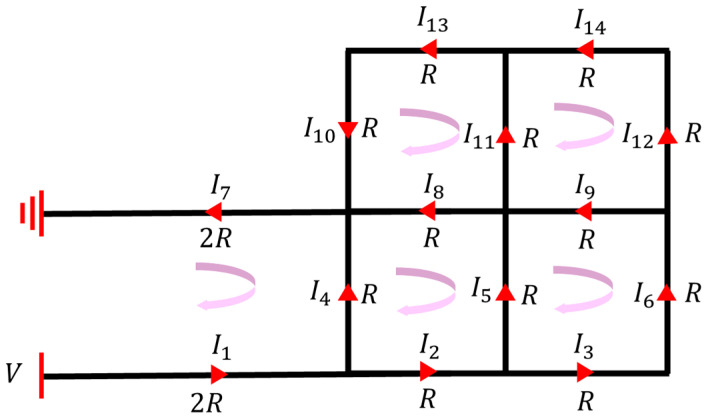
Application of KCL and KVL to the bilayer beam solar sail for current division. The red arrows represent the direction of currents across corresponding beams. The equivalent resistance of each bilayer grid beam was assumed to be R, while that of each bilayer supporting beam was 2R.

In a previous study, we simplified the beam structure and acquired an analytical solution to the temperature distribution via equivalent lumped models, which offered an efficient tool for the rapid evaluation and optimization of the concept design [39,40]. Similarly, given that the cross-sections and materials of each beam in the bilayer solar sail were identical, the equivalent thermal resistance per unit length of those beams was therefore the same. As such, the analytical modeling of the electro-thermal behavior of the bilayer solar sail in a high vacuum at ambient temperature started with the application of Kirchhoff’s voltage law (KVL) and Kirchhoff’s current law (KCL). As a result, the current division across the beam structure was obtained, as seen in Figure 3. $I_{i}$ (i = 1, 2, …, 14) is the current of each part, *V* is the voltage applied by the supporting beam, and *R* is the resistance of each part.

Specifically, the KCL equations could be written as

(3) $$\left\{\begin{array}{c} I_{1}{-I}_{2}{-I}_{4}=0\\ I_{2}{-I}_{3}{-I}_{5}=0\\ I_{3}{-I}_{6}=0\\ I_{4}{-I}_{7}+I_{8}+I_{10}=0\\ I_{5}{-I}_{8}+I_{9}{-I}_{11}=0\\ I_{6}{-I}_{9}{-I}_{12}=0\\ {-I}_{10}+I_{13}=0\\ I_{11}{-I}_{13}+I_{14}=0\\ I_{12}{-I}_{14}=0 \end{array}\right.$$

while the KVL equations were formulated as

(4) $$\left\{\begin{array}{c} V{-(I}_{1}+I_{7})\cdot 2R-I_{4}R=0\\ I_{4}{R-(I}_{2}+I_{5}+I_{8})R=0\\ I_{5}{R-(I}_{3}+I_{6}+I_{9})R=0\\ I_{8}{R-(I}_{10}+I_{11}+I_{13})R=0\\ (I_{9}+I_{11}){R-(I}_{12}+I_{14})R=0 \end{array}\right.$$

Hence, the 14 unknowns $I_{i}$(i = 1, 2, …, 14) were uniquely solved through the 14 independent equations in the equation sets of (3) and (4), that is

(5) $$\begin{array}{c} I_{1}=I_{7}=\frac{24}{113}\frac{V}{R}\\ I_{2}=\frac{7}{113}\frac{V}{R}\\ I_{3}=I_{6}=I_{10}=I_{13}=\frac{2}{113}\frac{V}{R}\\ I_{4}=\frac{17}{113}\frac{V}{R}\\ I_{5}=I_{8}=\frac{5}{113}\frac{V}{R}\\ I_{9}=I_{11}=I_{12}=I_{14}=\frac{1}{113}\frac{V}{R} \end{array}$$

**Figure 4:**
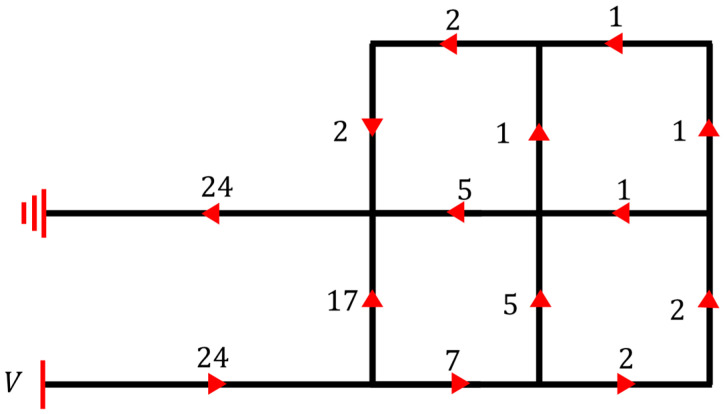
The current distribution across the beam structure (red arrows denote the current directions, while the numbers near the arrows represent the current division factors among different beams).

Finally, we obtained the ratios of currents flowing through different beam structures, as shown in Figure 4.

**Figure 5:**
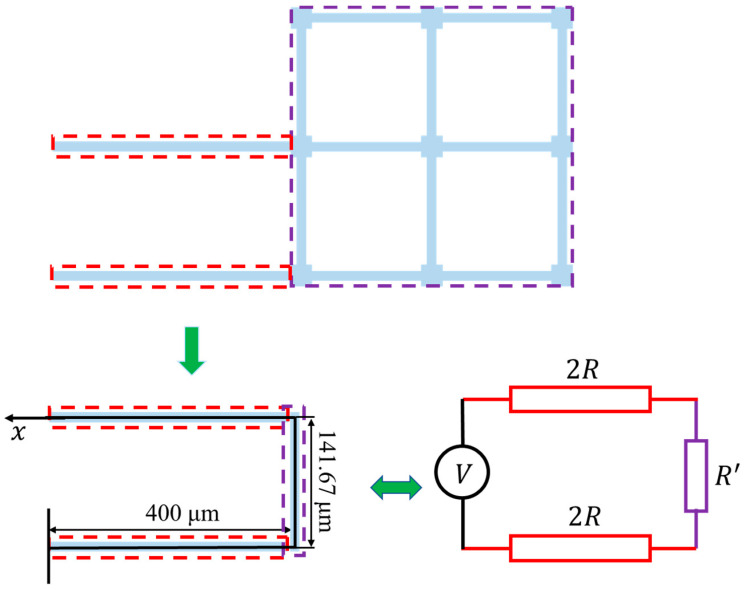
The equivalent U-shaped bilayer beam for the bilayer solar sail of the microrobotic ChipSail.

Since the cross-sections of each beam structure in the bilayer solar sail were identical, the resistance of each beam was proportional to its length. As such, the equivalent lumped model of such a bilayer solar sail was obtained based on the length of each beam. As a result, an equivalent 941.67 μm long U-shaped beam and its lumped model were acquired. The lumped modeling process of the bilayer solar sail is illustrated in Figure 5.

Given that the heat generation due to Joule heating should be equal to the heat dissipation via thermal conduction from the solar sail to the substrate (or heat sink), the relationship in such a steady state can be written as

(6) $$Q_{in}=Q_{out}$$

In particular, the one-dimensional heat flow equation for the equivalent U-shaped bilayer beam can be rewritten as

(7) $$\sum_{i=1}^{2}k_{i}w_{i}t\left[-{\left(\frac{dT}{dx}\right)}_{x}+{\left(\frac{dT}{dx}\right)}_{x+dx}\right]=\sum_{i=1}^{2}J_{i}^{2}\rho_{i}w_{i}t\cdot dx$$

where $k_{i}$ are the thermal conductivities, with i (i = 1, 2) denoting the Al and Ni_50_Ti_50_, respectively. $w_{i}$ represents effective widths of each material, while $T\left(x\right)$ is the temperature at distance x. Meanwhile, $J_{i}$ and $\varepsilon_{i}$ are the current densities and electrical resistivities, respectively, where $J_{i}$ can be calculated using

(8) $$J_{i}=\frac{V}{\varepsilon_{i}L}$$

Substituting Equation (8) into Equation (7), the following heat flow equation was obtained:

(9) $$\frac{d^{2}T}{dx^{2}}=-\frac{\left(\varepsilon_{1}+\varepsilon_{2}\right)V^{2}}{\left(k_{1}+k_{2}\right)\varepsilon_{1}\varepsilon_{2}L^{2}}$$

Since the ends of the supporting beam were assumed to have the same temperature as the substrate, the thermal boundary conditions were given as $T\left(0\right)=T\left(L\right)=T_{s}$ here.

Finally, the solution to Equation (9) is given as

(10) $$T\left(x\right)=-\frac{\left(\varepsilon_{1}+\varepsilon_{2}\right)V^{2}}{2\left(k_{1}+k_{2}\right)\varepsilon_{1}\varepsilon_{2}L^{2}}x^{2}+\frac{\left(\varepsilon_{1}+\varepsilon_{2}\right)V^{2}}{2\left(k_{1}+k_{2}\right)\varepsilon_{1}\varepsilon_{2}L}x+T_{s}$$

**Figure 6:**
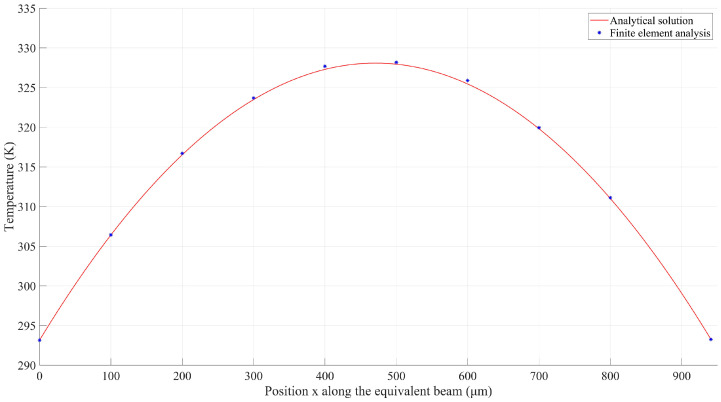
Comparison of temperature distributions between the analytical solution and finite element model when a voltage of 0.04 V was applied.

We compared the analytical solutions given by Equation (10) with the FEA results from Ansys under the same input voltage of 0.04 V. Since both supporting beams were connected to the anchored contact pads, the temperature of the left end of the beam was equal to that of the substrate, which was set to $T_{s}$ = 273.15 K. The temperature distribution along the equivalent U-shaped beam was plotted as discrete blue points in Figure 6, while the analytical solution to the mathematical model was drawn as a red solid line, which appears to be in good agreement with the FEA results. As such, the electro-thermal model was validated via the finite element method.

As seen in Figure 6, the highest temperature occurred at the center of the equivalent U-shaped beam, and a complete temperature distribution along the equivalent beam under certain applied voltages was necessary for further analytical thermo-mechanical modeling. As such, the analytical electro-thermal model, as well as finite element models, was employed to identify the relationship between the temperature peak and external electrical stimulus. It is worth noting that dT(0)/dx and dT(L)/dx represent the temperature gradients at the locations of x=0 and x=L, respectively, in the +x direction of the heat flow. Further, it can be safely concluded that dT(0)/dx>0, which implies that the temperature increases with increasing locations at x=0. Similarly, dT(L)/dx<0 reveals that the temperature dropped with increasing locations at x=L.

**Figure 7:**
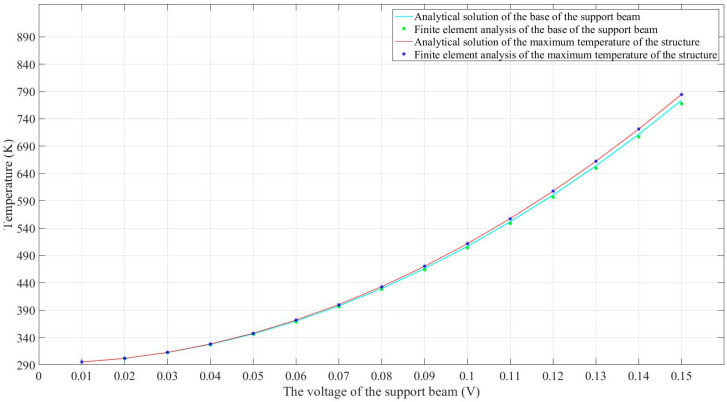
Comparison of analytical solutions and FEA results when different voltages were applied.

The solid red line in Figure 7 presents the analytical solutions of the maximum temperature with regard to different DC (direct current) voltages, while the discrete blue stars label the FEA results. According to the high consistency between the results, it can be safely concluded that the analytical model can function as an accurate tool for the prediction of the maximum temperature across the bilayer solar sail.

**Figure 8:**
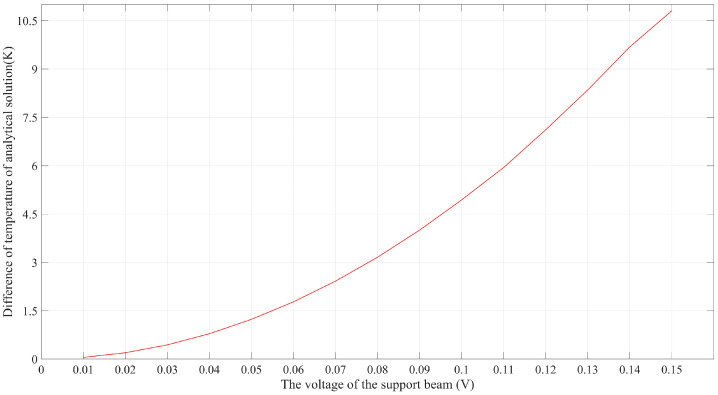
The temperature error between the temperature peak and the temperature at the distal end of supporting beams when different voltages were applied.

Another interesting phenomenon shown in Figure 6 is that the temperature distribution along the equivalent 141.67 μm long beam seemed to be uniform. This can be explained by the effect of thermal conduction along the supporting beams, which were two times longer than the grid beams. As a result, the distances between the heat sink and any points along the equivalent beam were similar, contributing to a nearly uniform thermal balance along the equivalent beam. To quantitatively evaluate the accuracy of the analytical model, the distal ends of supporting beams connected to the 141.67 μm long beam were selected as reference points. It can be clearly seen from Figure 6 that these two points shared the same temperature at any external DC voltage. The analytical solutions and FEA results at the distal end were acquired and are compared in Figure 7, which appears to show high consistency. Furthermore, the two solid lines in Figure 7 also imply that there was a very narrow temperature gap between the maximum temperature and the distal end temperature. The temperature difference between them regarding the applied voltages is plotted in Figure 8. This error was expected to be smaller when lower voltages were applied. As such, the peak temperature of the U-shaped beam could be safely and convincingly assumed to be the same as the temperature at the base, that is, the temperature along the 141.67 μm long beam was uniform.

### 3.2. Thermo-Mechanical Model

**Figure 9:**
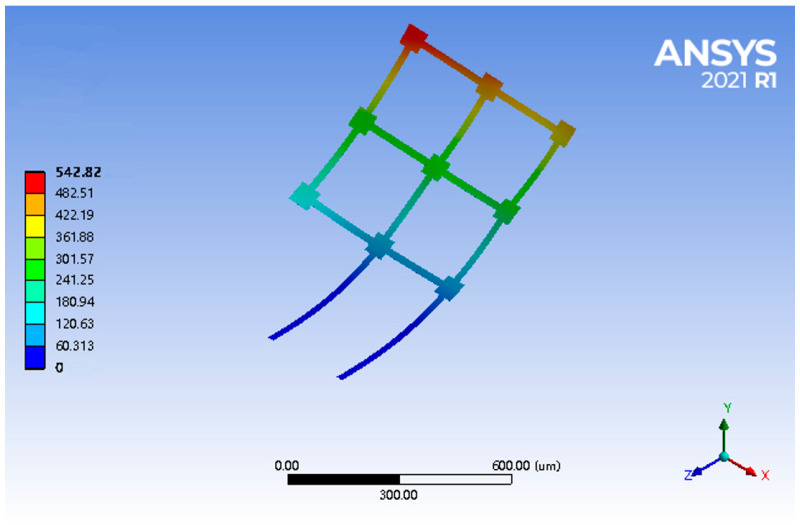
FEA results of the electro-thermo-mechanical deformation when a voltage of 0.04 V was applied between the supporting beams.

Based on the analytical electro-thermal model, thermo-mechanical modeling of the reconfiguration of the bilayer solar sail was accomplished and is discussed in this section. Prior to the analytical modeling, it was of great benefit to know the expected deformation under the electro-thermal actuation. Figure 9 shows the deformation of the bilayer solar sail structure under a voltage of 0.04 V. It was obvious that while the supporting beams exhibited arc-like bending, the remaining beams along the *Z*-axis could hardly deflect freely due to the effect of stiffness enhancement attributed to the beams along the *X*-axis. As such, the beam grid shown in Figure 1 was assumed to be a stiffness-enhanced beam plate that did not bend. Therefore, the reconfiguration of such a bilayer solar sail was solely dependent on the curvature along the supporting beam. Therefore, the modeling process assumed that when the structure shown in Figure 1 was deformed, only the support beams bent, while the beam mesh remained flat. As such, we could focus on the bending of the support beams. Specifically, we focused on the displacement along the *Y*-axis, and the analytical thermo-mechanical model was proposed and validated using the FEA method.

Given that the temperature along the supporting beams was changing with location x, the curvatures at different locations along the supporting beam were only determined by the local temperature, as seen in Figure 5. According to the Timoshenko model [41], the curvature at location x can be expressed by

(11) Cx=6αAl−αNiTiTx−Tsp+12tAl+tNiTi3p+12+pq+11pq+p2

**Figure 10:**
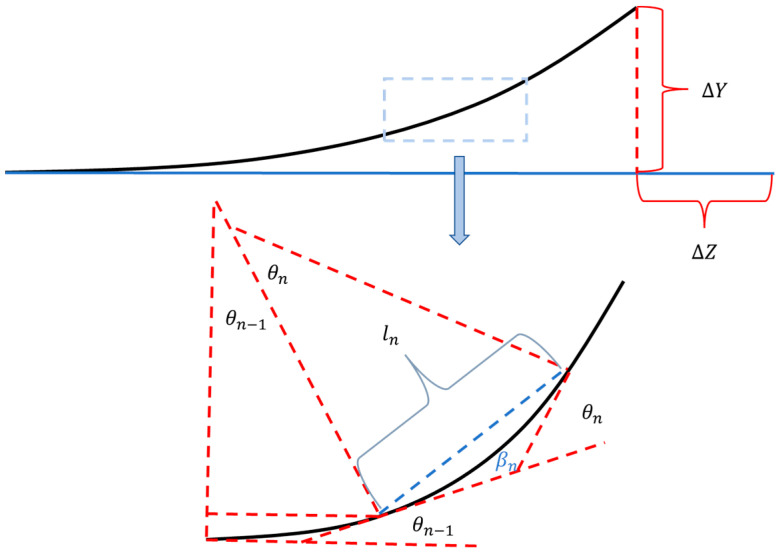
Differential method for determination of Y-deformation of the supporting beam. The out-of-plane deformed beam was discretized into multiple segments shown in the amplification area of the blue dashed box.

To acquire the deformation of the distal end of the supporting beam, this 400 μm long beam was divided into numerous small segments (differential method), and the Y-deformation of the distal end was a sum of each Y-deformation of these segments (see Figure 10). It is worth noting that any segment at x was deflected based on the accumulated bending angles of the segments located at positions smaller than x.

In the following analysis, the 400 μm long supporting beam was divided into N segments, and thus the equivalent curvature of the nth (n < m) segment could be solved using $C\left(\frac{nL}{N}\right)$. As such, the radius of the curvature could be written as

(12) $$r_{n}=\frac{1}{C\left(\frac{nL}{N}\right)}$$

The bending angle of the nth segment was obtained as

(13) $$\theta_{n}=\frac{C\left(\frac{nL}{N}\right)L}{N}$$

The angle between the nth chord (blue dashed line) and the corresponding tangent line was thus

(14) $$\beta_{n}=\frac{C\left(\frac{nL}{N}\right)L}{2N}$$

The length of the nth chord was formulated as

(15) $$l_{n}=\sqrt[{2}]{2{r_{n}}^{2}\left(1-cos\theta_{n}\right)}$$

The total tilt of the chord was

(16) $$\beta_{n,all}=\sum_{i=1}^{n-1}\theta_{n}+\beta_{n}$$

The total Y-deformation was

(17) $$Y_{all}=\sum_{n=1}^{N}l_{n}\beta_{n,all}$$

**Figure 11:**
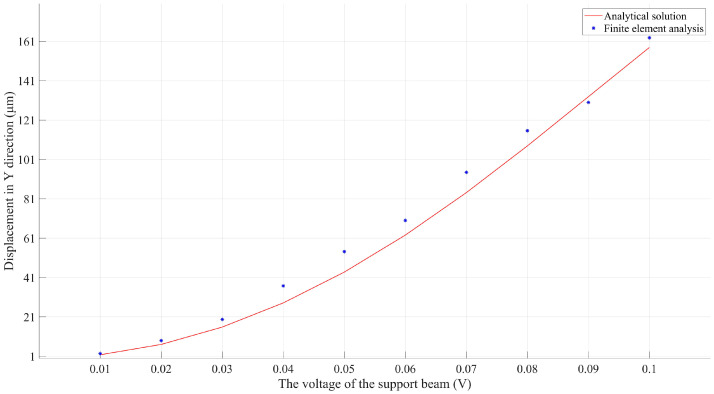
Comparison of the analytical solution and the FEA results for the Y-displacement of the microrobotic solar sail over a wide range of supply voltages.

Combining the thermo-mechanical model with the electro-thermal one, the analytical electro-thermo-mechanical model of the microrobotic bilayer solar sail in high vacuum was therefore obtained. Finite element models were built in the Ansys, and FEA results were collected by applying different DC voltages, which were compared with the analytical solution acquired by the analytical electro-thermo-mechanical model, as shown in Figure 11. The analytical solution appeared to be highly consistent with the FEA results regarding the Y-displacement over a wide range of supply voltages ranging from 0.01 V to 0.1 V. As such, the effectiveness and accuracy of the proposed analytical model for electro-thermo-electrical reconfiguration of such a microrobotic solar sail was demonstrated.

## 4. Fabrication and Experiment

**Figure 12:**
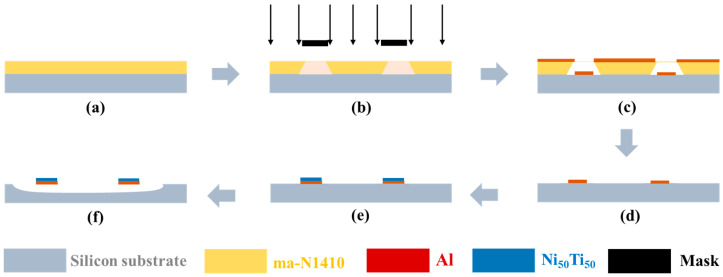
Fabrication process of the prototype of a solar sail beam structure. (**a**) ma-N1410 spin coating; (**b**) Photolithography; (**c**) Development and Al sputtering; (**d**) Lift-off for Al layer; (**e**) Sputtering Ni50Ti50 onto Al; (**f**) XeF2 Dry etch.

To demonstrate the active reconfigurability of the electrothermally driven microrobotic solar sails for the ChipSail, fabrication and in-situ characterization of a bilayer solar sail structure on wafer substrates were carried out. The fabrication process of the Al\Ni_50_Ti_50_ bilayer beam structure is shown in Figure 12, which was dry-etched and released from the substrate in the Xactix e2 XeF2 etch system [42].

**Figure 13:**
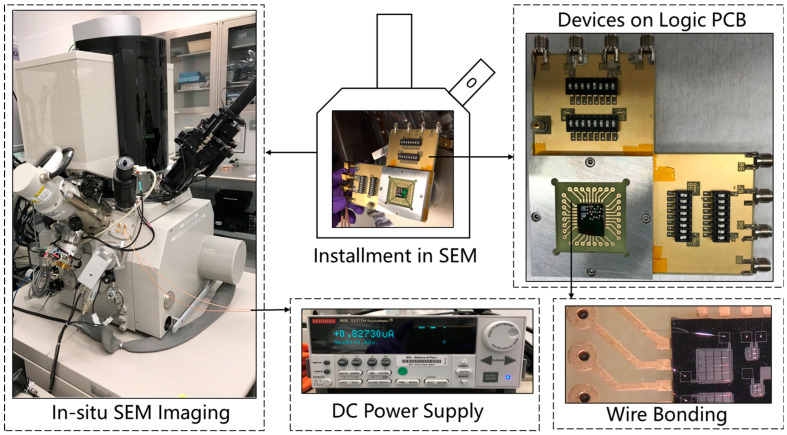
Experimental setup microstructures on the silicon chip for testing.

**Figure 14:**
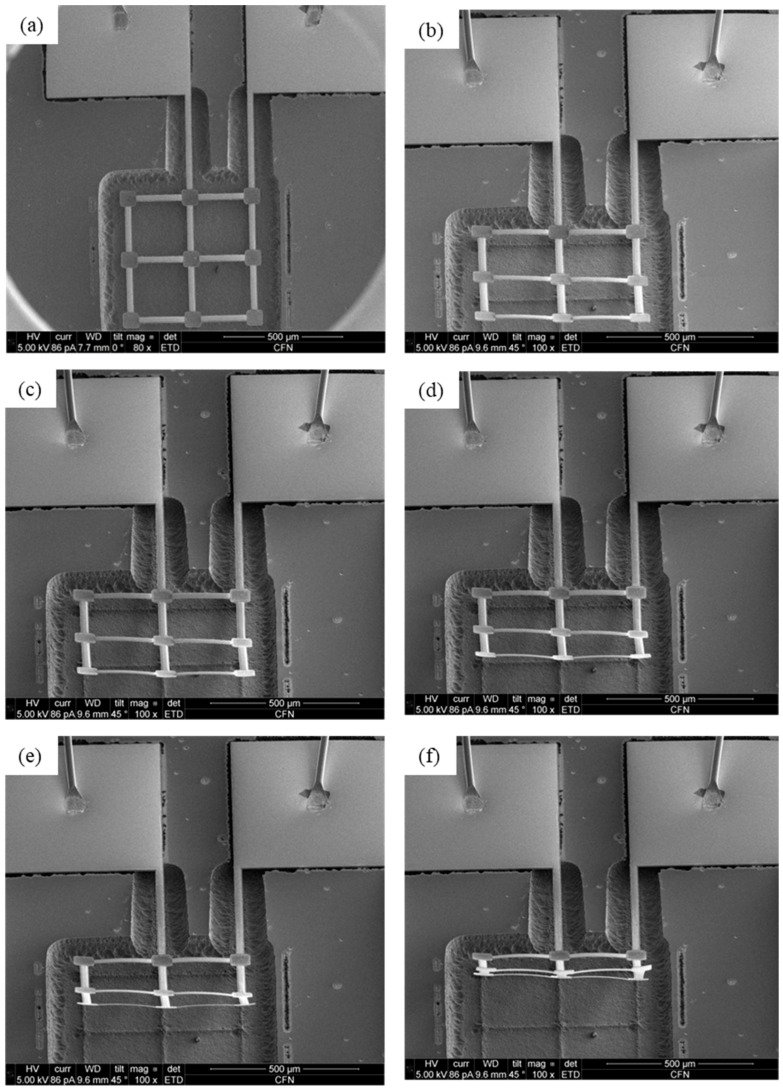
SEM images of bilayer solar sails under Joule heating with different driving voltages. (**a**) As-released bilayer solar sail without Joule heating; (**b**) SEM imaging with 45° tilt under 0 V; (**c**) Reconfiguration under 0.012 V; (**d**) Reconfiguration under 0.016 V; (**e**) Reconfiguration under 0.018 V; (**f**) Reconfiguration under 0.019 V.

In this work, we established a high vacuum surrounding using a scanning electron microscope (SEM) with an imaging system and an adjustable stage. The experimental setup for the in-situ test and displacement measurement of electrothermal actuation on the microrobotic device on silicon substrates is illustrated in Figure 13. The chip-scale (1 cm × 1 cm) silicon substrate (the gray square in Figure 13) with multiple bilayer beam structures was wire-bonded on a printed circuit board (the green square in Figure 13). Then, the printed circuit board was installed onto a logic board, which provided a variety of electrical signals to the bilayer beam structure via the wire that was bonded between the substrate and the printed circuit board. The stage of the SEM was tilted from 0° to 45° for easier observation of the active reconfiguration of the solar sail microstructures. The as-released microdevice without tilting is seen in Figure 14a, while that with a 45° tilt is shown in Figure 14b.

The geometric reconfigurations of the bilayer solar sail under the DC voltages of 0.012 V, 0.016 V, 0.018 V, and 0.019 V were imaged and are shown in Figure 14c–f, respectively. Based on the imaging principle and stage adjustment, the out-of-plane (or Y-displacement) could be quantitatively determined. As a result, the Y-displacements of the distal ends of the supporting beams were measured, and the experimental Y-displacements were further calculated to be 1 μm, 3 μm, 7 μm and 10 μm, respectively, which showed good agreement with the analytical solution shown in Figure 14. Hence, the viability of the analytical model of the electro-thermo-mechanical behaviors of this bilayer solar sail was demonstrated experimentally.

## 5. Discussion

A novel microrobotic bilayer solar sail structure with high area-to-mass ratios over 100 m^2^/kg was proposed for a next-generation solar sail spacecraft called ChipSails. Accelerations over 10^−4^ g are expected for photon propulsion of the ChipSail in geospace, enabling efficient attitude adjustment and orbital maneuvering. Given that the parameters of the area-to-mass ratio are dominated by the densities and thicknesses of different layers in the solar sail structure, the proper metallic combination of Al and Ni_50_Ti_50_ with significant differences in coefficients of thermal expansion was therefore chosen for the creation of a prototype of such bilayer solar sails. The conductivity of these materials ensured effective distributed Joule heating across the beam grid structure during thermal actuation, which contributed to active reconfiguration via the DC supply. Theoretical modeling, finite element analysis, and experimental validation were completed to demonstrate the reconfigurability of such electrothermally driven solar sails. Compared with the previous design of the ChipSail, the novel solar sail structure proposed in this study allows for further miniaturization of the conceptual satellite body based on flexible electronics in Figure 1. Furthermore, the conductive Al (0.3 μm)\Ni_50_Ti_50_ (0.3 μm) bilayer solar sail equipped with the chip-scale satellite body can function as microrobotic wings driven by the solar radiation pressure once the system is deployed in a space environment. The electro-thermal model for balanced thermal distribution across the solar sail structure was obtained using KVL, KCL, and the one-dimensional heat flow equation, while the thermo-mechanical model for structural deformation was given according to the Timoshenko model. The analytical solutions showed good agreement with the finite element results acquired using Ansys, which were further validated via fabrication and in-situ testing of a prototype of such solar sail structures. Therefore, the analytical model offers an efficient tool for the rapid evaluation and optimization of the structural design of the microrobotic solar sail for ChipSail systems.

However, it is worth noting that even though a high vacuum was enabled during the in-situ SEM imaging during the Joule heating process, the ultralow temperature in space (~0 K) was comparably difficult to be created physically in a ground-based lab. As such, the heat dissipation of the bilayer solar sail resulting from its thermal emission of radiation was not considered in this work. In addition, the initial deformation of the engineered bilayer solar sail prototype due to residual stress introduced during the fabrication and post-annealing processes was also neglected since there was no obvious initial bending detected, as seen in Figure 14a. Moreover, the electro-thermo-mechanical bending effect of the grid beam structure could be very complicated, and thus, the system was assumed to display no bending in this work. A more precise modeling of the grid beam bending with enhanced stiffness is yet to be established, which will be investigated further in future research.

Proposed ChipSail space systems exhibit promising applications, such as cleaning space debris from low Earth orbit and exploring the upper atmosphere. ChipSails can capture debris orbiting around the earth and then switch to drag sail mode, inducing an efficient deceleration and, consequently, the orbital decay of this space debris. In addition, cost-effective ChipSail formation flying enables remote and distributed sensing of upper atmosphere environments in the low Earth orbit. However, it is worth emphasizing that only debris with sizes comparable to the ChipSail can be efficiently removed, and the cleaning processes will also be inevitably influenced by the perturbations arising from atmospheric drag, the Earth’s magnetic field, and albedo, which will be fully taken into consideration in future work. Besides these, the payload for sensors tends to be severely limited due to the lightweight platform with high area-to-mass ratios. Therefore, good compromises between the capability and the budget of the ChipSail need to be made for specific space tasks.

We also envision that the microrobotic bilayer solar sail structure enabled by recent advances in microrobotics and microfabrication will play an important role in the development of next-generation solar sail spacecraft, such as ChipSails, for propellant-free solar sailing into the dark and deep space. The formation flight of considerable ChipSails in space allows for cost-effective, short-term space tasks, such as environmental probing and path-finding.

## 6. Conclusions

A novel microrobotic bilayer solar sail structure for a next-generation solar sail spacecraft named ChipSail was presented in this paper. Area-to-mass ratios over 100 m^2^/kg were realized via the development of solar sails for chip-scale satellite bodies using microfabrication processes, and the thickness constraint of each layer was at the sub-micrometer level. In particular, an Al (0.3 μm)\Ni_50_Ti_50_ (0.3 μm) bilayer beam grid structure was selected as a representative prototype for the feasibility demonstration of their reconfigurability via electro-thermal actuation. Electro-thermo-mechanical modeling of their behaviors via theoretical analysis and finite element method was accomplished in vacuum and ambient surroundings, which was then experimentally shown to be an effective approach for the rapid prediction and optimization of the structural design of such microrobotic bilayer solar sails. The current distribution was demonstrated by employing KCL and KVL, which implied various Joule heating powers across different bilayer beams, and thus, diverse balanced temperatures in certain environments. SEM imaging in a vacuum chamber revealed that out-of-plane displacements at the distal ends of supporting beams were up to 10 μm under a DC voltage application of 0.019 V, contributing to bending angles close to 90°. Active control of the solar sail’s bending angle ranging from 0° to 90° provides an effective solution to modify the solar radiation pressure distribution across the solar sail, which enables the desired attitude adjustment and orbital maneuvering of ChipSails for an exclusive and sustainable space service.

## Figures and Tables

**Table 1:** Mechanical and dimensional properties of the materials [33,34].

Properties	Bottom Al Layer	Upper Ni_50_Ti_50_ Layer
Electrical resistivity $\varepsilon_{i}$ (Ω⋅m)	2.65 × 10^−8^	82 × 10^−8^
Young’s modulus $E_{i}$ (Pa) Coefficient of thermal expansion $\alpha_{i}$ (K^−1^) Density $\rho_{i}$ (m^3^/kg) Length of the grid beam $L_{gi}$ (m) Length of the supporting beam $L_{i}$ (m) Width of the beam structure $w_{i}$ (m) Thickness of the beam structure $t_{i}$ (m)	70 × 10^9^ 23.1 × 10^−6^ 2.7 × 10^3^ 2 × 10^−4^ 4 × 10^−4^ 2 × 10^−5^ 3 × 10^−7^	56 × 10^9^ 11 × 10^−6^ 6.45 × 10^3^ 2 × 10^−4^ 4 × 10^−4^ 2 × 10^−5^ 3 × 10^−7^
Thermal conductivities $k_{i}$ (W/m⋅K)	205	18

## Data Availability

Data are available on reasonable request from the corresponding authors.

## Nomenclature

g	The local gravity in Earth’s orbit, m/s^2^
$m_{s}$	Mass of micro solar sails of the ChipSail, kg
$m_{c}$	Mass of the satellite body of the ChipSail, kg
m	Mass of the ChipSail, kg
$E_{Al}$	Young’s modulus of Al, Pa
$\rho_{Al}$	Density of Al thin film, 2.7 × 10^3^, kg/m^3^
$t_{Al}$	Thickness of Al thin film, m
$\alpha_{Al}$	Coefficient of thermal expansion of Al, K^−1^
$E_{NiTi}$	Young’s modulus of Ni_50_Ti_50_, Pa
$\rho_{NiTi}$	Density of Ni_50_Ti_50_ thin film, 6.45 × 10^3^ kg/m^3^
$t_{NiTi}$	Thickness of Ni_50_Ti_50_ thin film, m
$\alpha_{NiTi}$	Effective coefficient of thermal expansion of Ni_50_Ti_50_, K^−1^
$T_{0}$	Initial temperature in ambient condition, K
$T_{f}$	Final temperature via Joule heating, K
$T_{s}$	Balanced temperature without Joule heating in space, K
p	Thickness ratio of the Al layer to the Ni_50_Ti_50_ layer
q	Young’s moduli ratio of Al to Ni_50_Ti_50_
L	The equivalent length of a U-shaped beam, m
N	The number of U-shaped beam elements
$\theta_{n}$	The central angle of the arc, °
$\beta_{n}$	The angle between the string and the tangent line, °
$l_{n}$	The length of the string, m
$r_{n}$	The equivalent radius of the arc, m
